# Scurvy: hard to remember, easy to diagnose and treat^☆^^☆☆^

**DOI:** 10.1016/j.abd.2020.03.024

**Published:** 2021-01-31

**Authors:** Paulo Ricardo Martins Souza, Letícia Dupont, Felipe Eduardo Rodrigues

**Affiliations:** Dermatology Department, Santa Casa de Misericórdia de Porto Alegre, Porto Alegre, SR, Brazil

*Dear editor*,

Currently, scurvy is an uncommon disease, but it still exists, especially in groups at risk for hypovitaminosis. 1, 2, 3 Due to low suspicion, its clinical manifestations are often not well interpreted, leading to an extensive search for differential diagnoses ^1^. The dermatological findings are fundamental clues, especially perifollicular purpura, which appears to be found only in this disease ^2^. There are usually associated systemic symptoms, and bleeding is common ^2^. The prognosis is excellent, with clinical response in the first days of vitamin replacement 1, 2.

The authors report the case of a male patient, 63 years, hypertensive, diabetic, with chronic renal failure on hemodialysis for five years. He complained of asymptomatic lesions with progressive increase in the last two months, mainly in the lower limbs. In addition, he reported weakness, episodes of epistaxis, and reported food intake limited to sandwiches and other carbohydrates, denying consumption of fruits and vegetables. On physical examination, he had purpuric areas, plaques and isolated, mostly punctiform with perifollicular location, affecting the lower limbs (Figure 1, Figure 2), upper limbs, and back. Upon inspection of the oral cavity, no alterations were observed. Corkscrew hairs were observed at dermoscopy (Fig. 3). Extremely low plasma levels of ascorbic acid corroborated the diagnosis (0.08 mg/dL; reference value: 0.5–1.5 mg/dL). The anatomopathological exam showed folliculitis, perifolliculitis, and infundibular keratosis, common findings in scurvy. Days after oral supplementation of vitamin C, at a dose of 300 mg/day, resolution of the skin lesions was observed, in addition to improvement in asthenia and nasal bleeding. Gingival bleeding, historically the most classic manifestation of scurvy, was not observed in the present case, emphasizing that its absence does not exclude the diagnosis of the disease.

**Figure 1:**
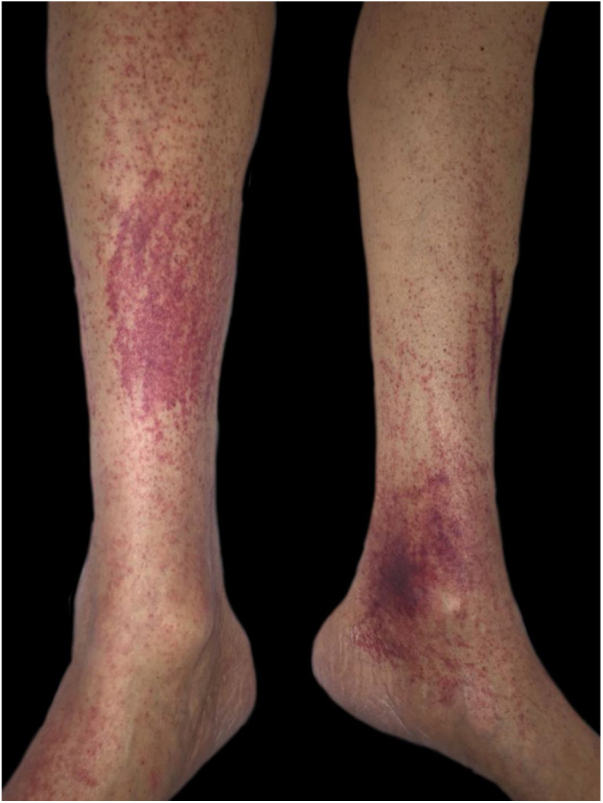
Purpuric areas in the lower limbs.

**Figure 2:**
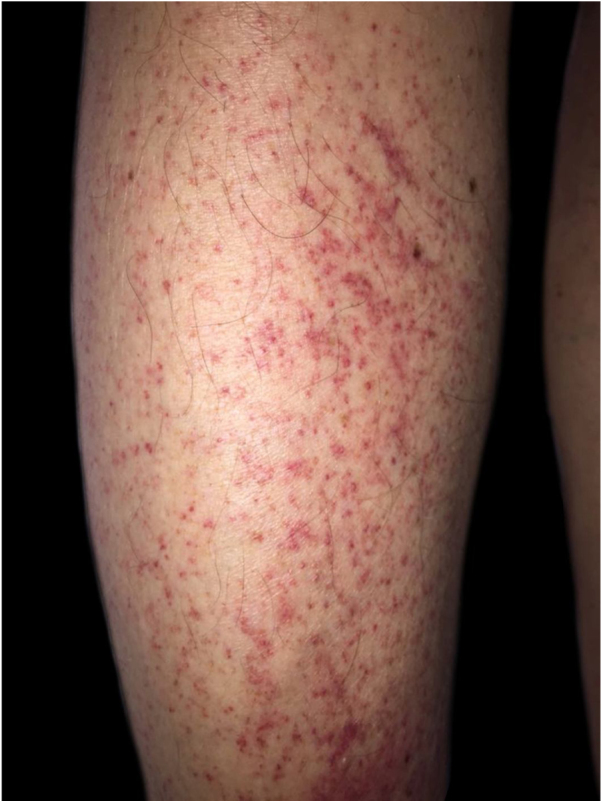
Perifollicular purpura.

**Figure 3:**
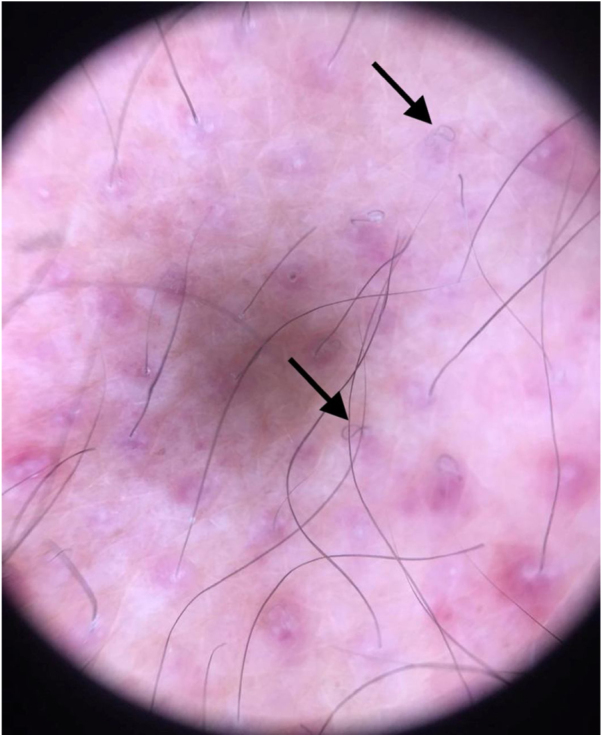
Dermoscopy: corkscrew hair.

Scurvy is caused by ascorbic acid (vitamin C) deficiency; vitamin C is found in fresh fruits and vegetables. 2, 3 Throughout history, scurvy was mostly diagnosed during the great Irish potato famine between 1845 and 1849, when the population of that country was reduced by 20% to 25%, the American civil war, and more recently the Afghanistan war in 2002. Although uncommon and remembered for its historical significance, scurvy is not a non-existent disease, especially in individuals with unusual diets, the elderly, alcoholics, patients with neoplasms or intestinal absorption disorders, and patients on hemodialysis. The kidneys reabsorb vitamin C and excrete it in the urine only when it exceeds the serum level; however, in dialysis this clearance is indiscriminate, increasing the risk of deficiency. ^3^

Ascorbic acid plays an important role in the formation of collagen and extracellular matrix, leukocyte function, iron absorption, folic acid metabolism, and other enzymatic processes. Anomalies in the collagen structure disrupt the integrity of the hair, connective tissue, and blood vessels, leading to the characteristic skin manifestations of scurvy. ^4^

The initial complaints, after one to three months of deficiency, are usually weakness, malaise, arthralgia, anorexia, and emotional liability. Capillary frailty predisposes to purpura, more frequent in the lower limbs, petechiae, ecchymosis, gingival bleeding, epistaxis, and bone hemorrhage. ^2^ Other dermatological findings include follicular hyperkeratosis and subungual hemorrhages ^4^. Dermoscopy reveals whitish hair follicles with “corkscrew” hair surrounded by a violaceous-hemorrhagic halo, with the whitish area corresponding to perifollicular fibrosis and the violet halo to the extravasation of red blood cells. ^5^

The diagnosis of scurvy is clinical, confirmed by low serum levels of vitamin C. Symptoms usually occur with plasma concentrations below 0.2 mg/dL. ^1^ The blood count usually reveals anemia and the inflammatory markers may be elevated. Biopsy mainly helps in distinguishing it from vasculitis, since the purpura in scurvy is of a non-inflammatory character. The classic histological pattern is perifollicular hemorrhage, irregularly shaped hair follicles with hyperkeratosis, and curved hair. ^2^

The prognosis for scurvy is excellent. ^1^ Ascorbic acid supplementation (100 mg three times a day) results in some symptomatic improvement on the first day and complete resolution of skin lesions within weeks ^4^.

Scurvy is probably underdiagnosed, although its manifestations are relevant to various medical specialties. It is remembered as a disease of ancient times, studied in high school and in history books, and not as a real diagnostic possibility. Attention should be paid to those patients with risk factors for nutritional deficiency, so that the classic findings of scurvy, an easily treatable disease, can be identified early.

## **Financial support**

None declared.

## Authors’ contributions

Paulo Ricardo Martins Souza: Approval of the final version of the manuscript, drafting and editing of the manuscript, effective participation in research orientation, intellectual participation in propaedeutic and/or therapeutic conduct of studied cases, critical review of the literature, critical review of the manuscript.

Letícia Dupont: Approval of the final version of the manuscript, design and planning of the study, drafting and editing of the manuscript, critical review of the literature, critical review of the manuscript.

Felipe Eduardo Rodrigues: Drafting and editing of the manuscript, critical review of the literature.

## Conflicts of interest

None declared.
